# Genomes of extinct hominins and human reproductive evolution

**DOI:** 10.17179/excli2022-5991

**Published:** 2023-03-22

**Authors:** Pallav Sengupta, Sulagna Dutta, Bhupender S. Chhikara

**Affiliations:** 1 Department of Biomedical Sciences, College of Medicine, Gulf Medical University, Ajman, UAE; 2 School of Medical Sciences, Bharath Institute of Higher Education and Research (BIHER), Tamil Nadu, India; 3 Medicinal Chemistry and Molecular Materials Lab, Department of Chemistry, Aditi Mahavidyalaya, University of Delhi, Delhi, India

## ⁯⁯⁯

The study of hominin genomes has provided insights into human evolution and reproductive functions. The hominin genome is the complete set of genetic material that is present in the cells of hominins, a group of primates that includes modern humans and their extinct relatives. It consists of approximately three billion base pairs of DNAs that are organized into 23 pairs of chromosomes (Yan and McCoy, 2020[13]). The genome contains information that determines a wide range of biological traits, such as physical characteristics, susceptibility to diseases, and cognitive abilities. It is inherited from one generation to the next and can be modified by mutations, genetic recombination, and other processes (Yan and McCoy, 2020[13]). Studying the hominin genome has been instrumental in understanding the evolution of humans and our relationships with other hominin species. The analysis of ancient hominin DNA has provided insights into the genetic diversity and population history of our ancestors, as well as their interactions with other species. The discovery of ancient DNA from extinct hominins, such as Neanderthals and Denisovans, has allowed scientists to reconstruct their genomes and compare them to those of modern humans. These comparisons have revealed that modern humans share genetic material with these extinct hominins, indicating that interbreeding occurred between these groups (Green et al., 2010[2]; Ko, 2016[5]).

One of the most significant findings from the study of hominin genomes is the evidence of interbreeding between early modern humans and Neanderthals. Genetic analysis has shown that Neanderthal DNA makes up approximately 1-4 % of the DNA in non-African populations (Veeramah and Hammer, 2014[12]). This interbreeding likely occurred when early modern humans migrated out of Africa and into Eurasia, where they encountered Neanderthals. The interbreeding could have had several effects on human evolution, including providing early modern humans with genetic adaptations that helped them survive in new environments (Ko, 2016[5]).

The study of hominin genomes has also shed light on the evolution of reproductive functions in humans. For example, genetic studies of the genomes of Neanderthals and modern humans have identified several genes that are involved in reproductive biology, including those that influence fertility, sperm motility, and the development of testes and ovaries (Greer et al., 2021[3]). Some of these genes include (a) DMRT1 (Doublesex and mab-3 related transcription factor 1) which is involved in the development of testes in males and the regulation of male fertility. Studies have found that Neanderthals and modern humans have different versions of this gene, suggesting differences in reproductive biology (Ramos and Antunes, 2022[9]), (b) ZP3 (zona pellucida glycoprotein 3) is involved in the formation of the zona pellucida, surrounding the oocyte and is essential for fertilization. While there has been limited research comparing the specific differences of ZP3 between Neanderthals and modern humans, it is found that Neanderthals and modern humans have different versions of this gene, which could have implications for fertilization and embryonic development (Hart et al., 2018[4]), (c) CATSPER (cation channels of sperm)1 encodes a protein that is involved in the regulation of sperm motility. Studies have found that the Neanderthal version of the CATSPER1 gene had several amino acid substitutions compared to the modern human version, which suggests that the Neanderthal version may have had a different functional impact on sperm. However, the exact implications of these differences are not yet fully understood (Green et al., 2010[2]), (d) AMH (anti-Mullerian hormone) gene is involved in the development of male reproductive organs and the regulation of female fertility. It is reported that Neanderthals and modern humans have different versions of this gene, which could have implications for reproductive biology. Studies have found that Neanderthals had higher levels of AMH than modern humans. This suggests that Neanderthals may have reached sexual maturity at an earlier age than modern humans, and that they may have had a shorter overall reproductive lifespan (Marín‐Arroyo and Sanz‐Royo, 2022[6]).

Some of these genes have undergone rapid evolution in humans, suggesting that they may have played a role in the adaptation of our species to different environments and lifestyles (Greer et al., 2021[3]). Another example, genetic analysis of the FOXP2 (forkhead box protein P2) gene, which is involved in speech and language, has shown that modern humans have a different version of the gene than Neanderthals and Denisovans (Mozzi et al., 2016[8]). This suggests that the evolution of language in modern humans was influenced by genetic changes that occurred after they diverged from their common ancestor with these extinct hominins. Another area where hominin genomes have provided insights into reproductive functions is the study of Y chromosomes. The Y chromosome is passed down paternally and can provide information about patrilineal ancestry. By comparing Y chromosomes from different populations, scientists have been able to trace the migration patterns of early humans. For example, analysis of Y chromosomes from modern humans and Neanderthals has shown that the two groups diverged from a common ancestor approximately 590,000 years ago (Skov et al., 2022[11]). The amount of information available is limited because the quality of DNA that has been recovered from Neanderthal fossils is generally poor. One study published in 2016 analyzed the Y chromosome of a 49,000-year-old male Neanderthal from El Sidrón, Spain, and found that the Neanderthal Y chromosome had several differences from the Y chromosome found in modern humans. Specifically, the study found that the Neanderthal Y chromosome had several mutations that are not found in the Y chromosomes of modern humans (Mendez et al., 2016[7]).

Moreover, the identification of genetic variants associated with fertility and reproduction in ancient hominin genomes has important implications for modern human health. For example, genetic studies of the genomes of Neanderthals and modern humans have identified variants associated with reduced fertility, which may have contributed to the extinction of some early human populations. A study published in 2016, examined the DNA of Neanderthals and found that they had a higher number of harmful mutations in genes related to male fertility compared to modern humans (Simons and Sella, 2016[10]). Additionally, a study published in the journal Proceedings of the Royal Society B: Biological Sciences in 2018 analyzed the size and shape of Neanderthal pelvic bones, which are important for childbirth, and found that they were narrower than those of modern humans (Betti and Manica, 2018[1]). This could indicate that Neanderthal women had a harder time giving birth, which could have contributed to reduced fertility. These findings highlight the importance of understanding the genetic basis of reproductive functions in both extinct and modern human populations, and may ultimately lead to the development of new treatments for infertility and other reproductive disorders (Skov et al., 2022[11]).

In conclusion, the study of hominin genomes has provided insights into human evolution and reproductive functions. The discovery of interbreeding between early modern humans and Neanderthals, as well as the existence of new hominin species like the Denisovans, has challenged our understanding of human origins and the process of speciation. The genetic analysis of FOXP2 and Y chromosomes has revealed how changes in our genome have influenced the evolution of language and migration patterns in early humans. Discovery of more fossils and ancient DNA would be critical in refining the understanding of human evolution and deciphering the existence of infertility like physiological ailments.

## Declaration

### Conflicts of interest

The authors declare no conflicts of interest.

### Acknowledgments

Not applicable.
